# Gene expression and metadata based identification of key genes for lung cancer, COPD, and IPF using machine learning and statistical models

**DOI:** 10.1371/journal.pone.0344666

**Published:** 2026-03-19

**Authors:** Mst. Farjana Yasmin, Md. Faruk Hosen, Md. Abul Basar, Anichur Rahman, Mahedi Hasan, Fahmid Al Farid, Hezerul Abdul Karim, Abu Saleh Musa Miah

**Affiliations:** 1 Department of Computing and Information System(CIS), Daffodil International University (DIU), Ashulia, Dhaka, Bangladesh; 2 Department of Computer Science and Engineering, Netrokona University, Netrokona, Bangladesh; 3 Department of Computer Science and Engineering, National Institute of Textile Engineering and Research (NITER), Constituent Institute of the University of Dhaka, Savar, Dhaka, Bangladesh; 4 School of Computing, Georgia Southern University, Statesboro, Georgia, United States of America; 5 Department of Information and Communication Technology (ICT), Mawlana Bhashani Science and Technology University (MBSTU), Santosh, Tangail, Bangladesh; 6 Faculty of Computer Science and Informatics, Berlin School of Business and Innovation Karl-Marx-Straße 97-99, Berlin, Germany; 7 Centre for Image and Vision Computing (CIVC), COE for Artificial Intelligence, Faculty of Artificial Intelligence and Engineering (FAIE), Multimedia University, Cyberjaya, Malaysia; 8 Department of Computer Science and Engineering, Rajshahi University, Rajshahi, Bangladesh; 9 Graduate Department of Computer Science and Engineering, The University of Aizu, Aizuwakamatsu, Japan; The First Hospital of Jilin University, CHINA

## Abstract

Lung cancer (LC) is one of the most prevalent and deadly cancers globally, presenting a major public health challenge. Patients with chronic obstructive pulmonary disease (COPD) and idiopathic pulmonary fibrosis (IPF) are at a significantly higher risk of developing lung cancer. Despite developments in research, the primary molecular pathways of many disorders remain poorly understood. The current study aimed to identify potential therapeutic genes for lung cancer (LC), chronic obstructive pulmonary disease (COPD), and idiopathic pulmonary fibrosis (IPF) through machine learning (ML) and bioinformatics methodologies. The differentially expressed genes (DEGs) were identified across three datasets utilising DESeq2 and limma, and the common genes among the DEGs from these datasets were subsequently selected. The protein-protein interaction (PPI) networks were generated utilising STRING, and major hub genes were discerned via topological analysis. The Key hub genes, such as ETS1, MSH2, RORA, and PMAIP1, were detected. The pathways named KEGG and cancer pathway studies were conducted to evaluate their contributions to disease processes. The research included network-based methodologies, including transcription factors, GO keywords, gene–miRNA relationships, and survival data analyses, to further narrow the list of differential genes linked to LC, COPD, and IPF. The metadata for hub genes was aggregated from prior studies to integrate earlier discoveries. In the end, four key candidate genes (ETS1, MSH2, RORA, and PMAIP1) were found by intersecting the common differentially expressed genes, hub genes, major module genes, and meta-hub genes. The outcomes present a solid framework for subsequent research and therapy strategies for LC, COPD, and IPF. The potential drug compounds targeting the identified key genes are proposed, offering new avenues for the development of treatment.

## 1 Introduction

Globally, lung cancer is the leading cause of cancer-related mortality. Oncogene mutations are generally responsible for the development of lung cancer, as they cause aberrant cell proliferation that leads to the formation of lung tumours [1]. Among all cancer types, lung cancer continues to rank among those with the highest incidence. Based on histopathological features, it is categorised into two categories: non-small cell lung cancer and small cell lung cancer [2]. Lung cancers caused by smoking are still common, even if smoking rates are dropping. As of 2023, lung cancer among nonsmokers ranks seventh worldwide in terms of cancer-related fatalities; it predominantly affects Asian and female individuals [3]. Males are approximately twice as likely to develop lung cancer compared to females, and both the incidence and death rates are three to four times higher in developed nations than in developing nations [4]. Given the link between increased smoking and the development of lung cancer, tobacco control measures may have a major influence on lung cancer rates in the future. It is estimated that in 2030, there will be ten million deaths every year due to lung cancer [5]. Various research studies have found the onset of diseases like LC, IPF, and COPD to be common. However, the simultaneous onset of these three diseases amplifies the risks and complications faced. This paper proposes to research these three diseases individually and try to explore whether there are any links among them or not. Patients with a single disease are less susceptible to lung cancer than patients with multiple diseases. Unravelling the connections between different diseases and different genes, as well as various interactions among the genes for these diseases, is the essence of this research work. However, it is important to notice that airway obstruction with inefficient inflammatory reactions against environmental toxins is indicative of COPD, which is quite treatable [6]. Globally, chronic obstructive pulmonary disease (COPD) is ranked #4 because of smoking [7,8]. Communities worldwide also have significant costs because of societal implications of this disease [9–11]. The presence of comorbidities such as chronic obstructive pulmonary disease (COPD) and other health issues like cardiovascular disease, along with morbidity/mortality related to it, is already explainable because [12–14], further complicating matters. [15,16]. The additional risk for developing lung cancer may be elevated for patients with COPD [17]. The relative risk of developing lung cancer was five times greater in cigarette smokers who had chronic obstructive pulmonary disease (COPD) than in those who had sufficient lung function [18]. Reduced lung function is a significant risk factor for lung cancer. Forced expiratory volume in one second (FEV1) is one of many respiratory risks associated with smoking, which could be implicated in lung cancer and COPD development. Another risk is Chronic Obstructive Pulmonary Disease (COPD) [19]. In addition, smoking and becoming older are risk factors for the chronic lung disease known as idiopathic pulmonary fibrosis (IPF). A complex interplay of genetic, epigenetic, immunologic, and environmental variables characterises idiopathic pulmonary fibrosis (IPF), a debilitating lung disease that is on the rise [20,21]. This condition is characterised by the fact that it is becoming more prevalent. Three distinct types of disease progression have been proposed: illness that is generally stable with acute exacerbations in between; disease that is rapidly progressive; and disease that is slowly progressive [22–24]. A recent study suggests that interstitial pulmonary fibrosis (IPF) is a highly polygenic illness, meaning that there are a number of changes that are connected to susceptibility to the disease [25,26]. Furthermore, IPF is linked to restrictions in lung function testing [27]. The tissue encircling the alveoli, or the lung’s air sacs, may sustain damage as a result of this severe type of illness [28]. Since people with IPF may also acquire lung cancer, IPF is regarded as a progressive interstitial lung disease with features resembling malignancy. The epidemiological data indicate that the risk of lung cancer among individuals with IPF is roughly 3.34 times greater than that observed in the general population [29]. Although LC is thought to be an advanced form of IPF, research has shown mixed findings about the precise histological forms of lung cancer associated with IPF [30].

**Fig 1:**
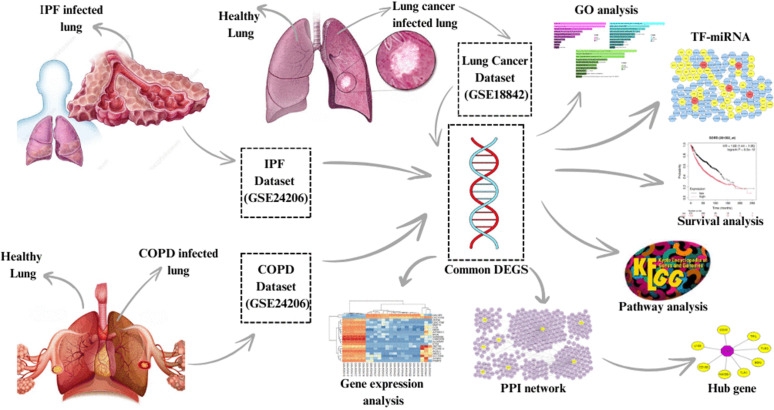
The diagram depicts the process of research, analysis, and the information flow. Sample information was extracted following the examination of publicly available datasets: GSE76925, GSE24206, and GSE18842. Both healthy and diseased cell populations were represented within the sample data. The samples obtained from 11 IPF-affected patients undergoing lung transplantation or diagnostic surgical biopsy are included in the GSE24206 dataset. There are 91 samples of NSCLC in the GSE18842 collection. In the GSE76925 cohort, including 40 as control subjects and 111 infected patients, 214 genes were found to be differentially expressed. From these datasets, the analysis conducted in R facilitated the identification of common DEGs. These common genes were then used to study KEGG pathways, pharmacological signatures, and Protein-Protein Interactions (PPI) networks.

According to a recently released genome sequencing study, IPF and LC have several somatic mutations in common [31]. The link between IPF and lung cancer is supported by extensive epidemiological surveys. According to a systematic review, it is suggested that IPF may have the same genetic variation as lung cancer [32]. According to a great study by Tzouvelekis et al., IPF and LC may be caused by similar genetic, epigenetic, and cellular mechanisms, such as abnormal activation of signalling pathways including PI3K/AKT and Wnt *β*-catenin, which results in hyperproliferation and metaplasia [33]. To clarify the connection among LC, COPD, and IPF and investigate novel avenues for disease treatment, this study used gene-based research. High-throughput techniques are becoming increasingly popular, especially for analysing microarray data and information extracted from expression datasets of this kind. Eight common genes were identified as shared among the GSE24206, GSE18842, and GSE76925 datasets following gene analysis. Since the Protein-Protein Interaction (PPI) network is a crucial component of the current investigation, it will subsequently be the subject of the following examination. To visualise the relationships between Differentially Expressed Genes (DEGs), a PPI network is constructed, and a degree topological method is used to identify and rank hub genes. It has been standard practice in bioinformatics research to combine related DEGs to identify putative medicinal drugs based on these genes. Protein–protein interaction networks represent fundamental resources for exploring the biological processes involved in disease development, biological functions, and the development of medication. Nevertheless, due to its complexity, decoding the network is difficult [34]. Additionally, this study includes the analysis of frequent DEGs, with a focus on gene ontology (GO) and other biological pathways. To help biological researchers, microarray data, including molecular information, can be computationally evaluated. The main aims of this study will be finding the related biomarkers according to gene-based analysis results, as well as exploring the molecular relationships between Lung Cancer, COPD, and IPF. The key point of finding the genes associated with the discussed diseases would be the application of differentially expressed genes (DEGs). Subsequently, KEGG pathway analysis is performed to investigate the core biological functions and pathways involved. The final step involves proposing therapeutic candidates based on the shared DEGs identified among IPF, COPD, and LC following hub gene selection. The approach used in this work was depicted in Fig 1.

## 2 Materials and methods

### 2.1 Details informations of datasets

The GSE24206, GSE18842, and GSE76925 datasets were created using the information from the GEO database [35]. In the GSE24206 data series, collected 11 samples that causes IPF and 6 control samples. The GSE24206 comprises lung tissue samples from IPF patients undergoing lung transplantation or diagnostic surgical biopsy, diagnosed using standard clinical, radiological (HRCT), and histopathological criteria. Lung tissues from healthy transplant donors were used as controls; IPF severity staging data were not uniformly available. The GSE24206 data set was analyzed using the GPL570 platforms. The GSE18842 collection included a total of 91 non-small cell lung cancer (NSCLC) samples. Among of them, 46 cases were cancerous and 45 were normal. The GSE18842 dataset used in this study primarily provides gene expression profiles derived from tumor and matched normal lung tissues, without detailed annotation of somatic mutations (e.g., EGFR, KRAS, TP53) or TNM staging information. The GSE18842 dataset was analyzed using the GPL570 platforms. The GSE76925 includes lung tissue samples from clinically diagnosed COPD patients and non-smoker controls, classified based on clinical diagnosis and smoking status. Detailed severity information (e.g., GOLD stage or FEV1) was not uniformly available; therefore, analyses focused on molecular signatures associated with COPD presence rather than disease severity.The total 214 genes had differential expression in the GSE76925 sample, which comprised 111 COPD patients and 40 nonsmokers as controls. The GSE76925 dataset was analyzed using the GPL10558 platforms.

### 2.2 DEGs detection and identification of shared across lung cancer, COPD, and IPF

A gene is considered differentially expressed when there is a statistically significant difference between multiple test conditions at the transcription level [36]. The main goal of this study is to detect DEGs in the datasets GSE18842, GSE24206, and GSE76925. The most frequently occurring DEGs may be detected through analyses conducted with R. Two threshold criteria were used for each dataset in order to identify statistically significant DEGs: a p-value of 0.01 and 1 logFC > 1.7 for up-regulated data, and −1.7 logFC > -1 for down-regulated data [37]. The shared DEGs of GSE24206, GSE18842, and GSE76925 were determined online using the Venn VENN analysis tool.

### 2.3 ML-based gene selections

DNA microarray technology enables the simultaneous measurement of expression levels for thousands of genes within a single experiment. Analysing gene expression data is crucial across various biological research domains to extract meaningful insights, [38]. The first step is to find useful information in these big, complicated data sets. This task is what techniques in feature selection aim to solve by identifying informative marker genes, which will in turn increase classification performance by eliminating irrelevant features [38–40].

#### 2.3.1 First-step gene selection using mRMR.

Differentially expressed genes (DEGs) are generally detected through empirical Bayes moderated tests incorporating false discovery rate (FDR) adjustments. This method works well, but it doesn’t deal with gene redundancy. The mRMR improves feature selection in terms of optimising relevance and reducing redundancy. The mRMR is commonly utilised in the fields of machine learning and multiomics studies as an approach that upgrades the quality of the gene subsets that are selected. citebib41.

The mathematical representation of the mRMR algorithm is given below [41]. Let *Ψ*, $\Psi_{s}\;$, and $\Psi_{t}\;$ denote the set of all features (all genes from *β* which were chosen using differential expression analysis), is the set of features chosen, and the set of features to be chosen, respectively. The relevance (*D*) of a feature *f* from $\Psi_{t}\;$ with the target tissue or cell type *t* is measured using mutual information (*I*):


D=I(f,t)  (1)

The redundancy (*R*) of a feature *f* with the features already selected in $\Psi_{s}\;$ is defined as:


$$R=\frac{1}{m}\sum_{f_{i}\in \Psi_{s}}I(f,f_{i})\;$$ (2)

where the feature numbers are represents *m* in $\Psi_{s}\;$. The objective is to select a feature *f*_*j*_ from $\Psi_{t}\;$ that maximizes the relevance *D* while minimizing the redundancy *R*. This optimization problem can be expressed as:


$$\max_{f_{j}\in \Phi_{t}}\left[D(f_{j},t)-R(f_{j},\Psi_{s})\right]\;$$ (3)

After *n* iterations of evaluation, all features (*Ψ*) are ranked to produce a reordered feature list $\alpha^{\prime }\;$ of the new gene expression matrix of $\beta^{\prime }\;$ as follows:


$$\alpha^{\prime }=\{f_{1}^{\prime },f_{2}^{\prime },\ldots ,f_{i}^{\prime },\ldots ,f_{N}^{\prime }\}\;$$ (4)

In this case, the importance of the index *i* is related to its relevance to the target as well as redundancy in the features. The lower the value of the index *i* for a feature, the more discriminative it is, hence assigning a higher rank to feature *f*_*i*_. The most relevant genes are identified in the initial step for gene selection from the list containing the features $\alpha^{\prime }\;$.

#### 2.3.2 Second-step gene selection using SVM-RFE.

SVM-RFE is a type of supervised feature selection technique wherein genes are ranked based on the iterative development of the SVM model with the successive removal of the least significant features. This process helps identify the gene that has the strongest impact on gene expression related to cancer, thereby improving accuracy and interpretability. SVM-RFE is handy for high-dimensional data [42].

The mathematical formulations underlying the SVM-RFE algorithm are outlined below [43]:

#### 2.3.3 1) SVM decision function.

The SVM classifier constructs a decision function to separate two classes by identifying an optimal hyperplane *f*(*x*), defined as:


$$f(x)=\text{sign}\left(\sum_{i=1}^{n}\alpha_{i}y_{i}K(x_{i},x)+b\right)\;$$ (5)

where:

- *x*: Input feature vector,
- $\alpha_{i}\;$: Lagrange multipliers,
- *y*_*i*_: Class labels (+1 or −1),
- *K*(*x*_*i*_, *x*): Kernel function (e.g., linear, polynomial, Gaussian),
- *b*: Bias term.

#### 2.3.4 2) Feature importance.

The importance of each feature *j* is calculated based on the squared weight assigned by the SVM:


$$w_{j}^{2}={\left(\sum_{i=1}^{n}\alpha_{i}y_{i}x_{ij}\right)}^{2}\;$$ (6)

where the value of feature *j* is represents by *x_ij_* for the *i* -th sample. Features with lower $w_{j}^{2}\;$ contribute less to the classification boundary.

#### 2.3.5 3) Recursive elimination process.

SVM-RFE recursively discards less relevant or non-informative features. In each iteration, the SVM model is trained on the current feature set, and the importance scores $w_{j}^{2}\;$ are calculated for all features. The feature with the smallest score is eliminated, and the process is repeated until a predefined number of features remains.

#### 2.3.6 4) Objective function.

The SVM training process aims to minimize the following objective function:


$$\min\frac{1}{2}\|w{\|}^{2}+C\sum_{i=1}^{n}\xi_{i}\;$$ (7)

subject to:


$$y_{i}\left(w\cdot x_{i}+b\right)\geq 1-\xi_{i},\;\xi_{i}\geq 0\;$$ (8)

Here, $\|w{\|}^{2}\;$ serves as a regularization term to maximize the margin, *C* is a penalty parameter controlling the trade-off between margin size and classification error, and $\xi_{i}\;$ are slack variables that allow soft-margin violations.

SVM-RFE ranks genes based on their influence on the decision boundary and progressively eliminates less important genes. In this second-step feature selection phase, we applied SVM-RFE to the refined gene set $\alpha^{\prime }\;$, and retrieved a subset of the most informative and discriminative genes, denoted as $\alpha^{\prime \prime }\;$. Genes from the list $\alpha^{\prime \prime }\;$ were considered as ML-DEGs.

### 2.4 Carrying out gene ontology and pathway enrichment analysis

A crucial technique that identifies gene sets associated with specific chromosomal locations and functional importance is called gene set enrichment analysis. Understanding metabolic pathways and gene annotations is facilitated by the use of KEGG pathways. An online tool called Enrichr made it easier to look at genes that were found to be similar. The terms used in Gene Ontology (GO) [44,45] to explain the molecular activities and functions of genes are grouped. There are three main groups: biological processes (BP), cellular components (CC), and molecular functions (MF) [46]. To streamline and enrich the analysis of the pathways, the study incorporated WikiPathways [47], Reactome [48], and BioCarta [49] in addition to KEGG. The KEGG pathways [50,51] are well known for helping us understand metabolic pathways. The online tool Enrichr [52] has combined information from these databases to look at the biological processes and pathways that involve DEGs that are shared between lung diseases such as IPF, COPD, and lung cancer. In this method, an enrichr was used to determine the routes and biological processes. It is especially helpful to use KEGG pathways to map metabolic processes, which are very important in genetic studies. An established test (P value < 0.05) was used in establishing the significance of paths identified.

### 2.5 Examining the PPI network and hub gene identification

It has universally been recognised that the PPI interaction is the study hotspot in cellular biology and that the study of PPI is supposed to be an essential prerequisite for systems biology. It clarifies how proteins carry out their biological roles [53]. They are molecular bonds formed by hydrophobic, electrostatic, and biochemical variables between two or more proteins. PPI networks provide valuable insights into how proteins function. We created the networks with the use of NetworkAnalyst, based on physical interactions of DEGs found in the database named string [54]. The network structure becomes more transparent and visualised by the application of some programs like Cytoscape [https://cytoscape.org/]. By using PPI analysis exploiting topological features, it was identified that the proteins interact strongly with a degree greater than 13° and act as hub proteins. In such a network, finding the key components, called central genes, is highly important to understand complex biological processes. The degree topological method was used with Cytoscape [55] and the CytoHubba [56] tool to examine PPI networks and describe gene connections. The MCC technique typically detects the central/hub genes in the network, while CytoHubba provides the other tools. The MCC technique in CytoHubba was employed to identify the top five hub genes in the network. The top five hub genes are crucial in the formation of the network. The other features in CytoHubba allow us to determine the shortest paths between the hub genes; this provided us with further details about the functions of the hub genes in the network. The technique used in this study allows us to learn more about the other vital genes and their interactions in the biological processes.

### 2.6 Validation of the identified hub genes

#### 2.6.1 Discriminative power evaluation.

The public GEO datasets GSE33532 and GSE40791 were used to verify the discriminant power of the identified hub genes. The logistic regression method for tumour vs. normal discrimination [57] and the ROC curve with the AUC metric (58) were employed to assess the discriminant abilities. A leave-one-out cross-validation (LOOCV) framework was used for evaluation to reduce bias and improve the dependability of the findings [41].

#### 2.6.2 Survival analysis.

Our analysis focuses on transcriptomic alterations common across lung diseases, rather than mutation-driven oncogenic subtypes. Survival analysis was performed using expression-based stratification, which partially captures disease aggressiveness despite the absence of stage-level metadata. We looked at how long hub genes that are found in lung cancer, COPD, and idiopathic pulmonary fibrosis (IPF) stay alive in this work. Based on network analysis, MSH2, ETS1, RORA, SORD, CCNL1, CFH, NEDD9, and PMAIP1 were selected as the principal hub genes.We utilized gene expression and clinical information in identifying and predicting the gene expression profiles that corresponded with these ailments. The Cox proportional hazards (PH) model and the product limit (PL) estimator helped us determine the survival function both for the altered group and the regular group. There were two parts to the Cox PH regression analysis: the univariate part and the multivariate part. The univariate analysis assessed the impact of each hub gene individually, while the multivariate analysis explored the combined effect of multiple genes. To ascertain the relevance of each gene in predicting patient survival, we computed the predicted coefficients (β, hazard ratios (HR), and corresponding p-values. The value p < 0.05 indicated that a gene was significant. After that, the PL estimator was used to create survival curves for the most important genes, which contrasted the survival rates of the groups with changed and normal expression.

### 2.7 Finding the miRNAs AND Transcriptions factors that work with the common DEG signatures

During the process of genetic transcription, transcription factors (TFs) regulate the expression of genes. We found transcription factors from the JASPAR database [58] that bind to the same differentially expressed genes (DEGs) and show that the network is topologically stable using NetworkAnalyst. The JASPAR database contains TF profiles for many different species, and NetworkAnalyst has a lot of biological information and gene expression analyses [59]. It was also possible to look into how miRNAs target gene interactions using the Tarbase and mirTarbase databases [60]. We collected miRNAs linked to common Differentially Expressed Genes (DEGs) for topological analysis by using NetworkAnalyst to strengthen the links between miRNAs and genes. This tool, Cytoscape, made it easier to see networks of TF genes and miRNA genes. This helped with high-degree miRNA filtering and the creation of new, more accurate biological ideas. Also, the RegNetwork resource provided the coregulation interactions between TF and miRNA, which aided in understanding the control mechanisms of DEGs from transcription to post-transcription. NetworkAnalyst was used to create a coregulatory network with both TF and miRNA regulatory parts.

### 2.8 Identification of therapeutic drugs

Finding therapeutic molecules is the main goal of the current study. These molecules are needed to treat diseases like IPF, lung cancer, and COPD. DSigDB [61], which has 22,527 gene sets, helps with predicting drug compounds based on gene expression patterns. The main way to get to DSigDB is through Enrichr, which lets you do a lot of enrichment analysis and see how genes work. You can guess protein-drug interactions (PDI) and look for possible drug molecules linked to the diseases on the list by using Enrichr’s access to DSigDB. This way of doing things shows how important it is to use gene expression data when finding new drugs and making new medicines.

## 3 Results

### 3.1 Experimental settings

R (version 4.1.2) was used for all statistical analyses. An Intel(R) Core (TM) i5 processor running the Windows operating system was used to carry out the calculations.

#### 3.1.1 Dataset information.

The collection of gene sets related to the disorders under consideration is obtained from the NCBI database. After processing and organization, 693 genes have been linked to IPF; of these, 310 have been linked to up-regulation and 383 to down-regulation; 547 have been linked to COPD; of these, 497 have been linked to up-regulation and 50 to down-regulation; and 2338 have been linked to lung cancer; of these, 1064 have been linked to up-regulation and 1274 to down-regulation in humans. The weight of each gene determines how they are ordered in ascending order. The expression levels of the identified vulnerable genes are detailed in Table 1.

**Table 1:** Collection of differentially expressed genes (DEGs) associated with the targeted diseases obtained from the GEO datasets in the NCBI database. The DEGs are arranged in ascending order based on their assigned weights.

Disease Name	GEO accession	GEO platform	Total DEGs	Up-regulated	Down-regulated
Idiopathic Pulmonary Fibrosis (IPF)	GSE24206	GPL570	693	310	383
Chronic Obstructive Pulmonary Disease (COPD)	GSE76925	GPL570	547	497	50
Lung Cancer	GSE18842	GPL10558	2338	1064	1274

#### 3.1.2 Analysis of LC, IPF, AND COPD gene expression.

**Fig 2:**
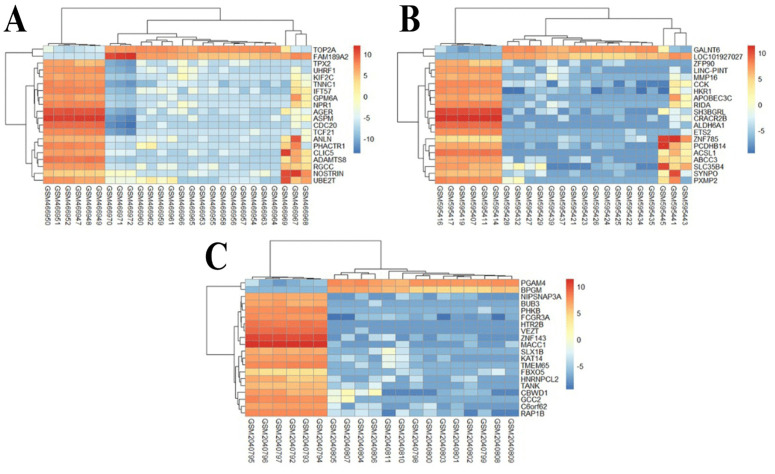
(A) Gene expression profiles of IPF-affected tissues were analysed using 23 samples selected from the GSE24206 dataset, with a focus on the top 20 genes. **(B)** 23 samples were chosen from the GSE76925 dataset, and the expression of the top 20 genes in tissues impacted by COPD was evaluated. **(C)** Using 26 samples selected from the GSE18842 dataset, the expression of the top 20 genes in lung cancer-affected tissues was examined. The distinct expression patterns of these important genes in each of the three diseases are graphically depicted in this heatmap. This kind of comparative research helps distinguish between distinct and overlapping genetic markers.

**Fig 3:**
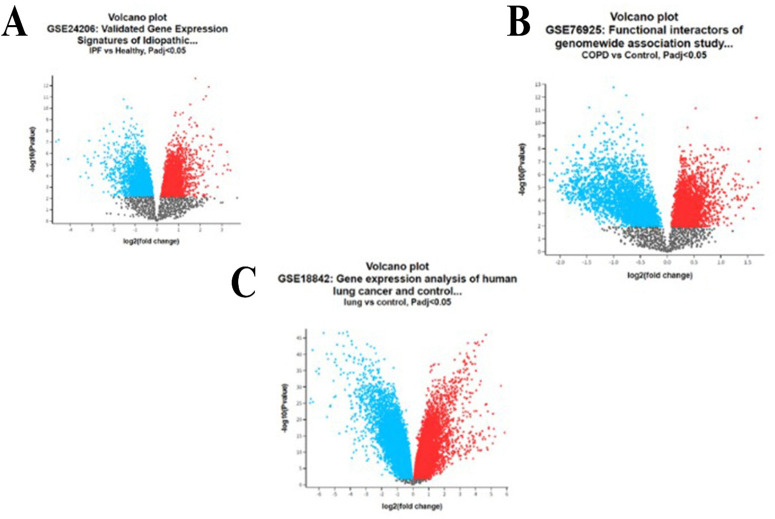
An extensive summary of the alterations in gene expression in each disease state is given by these maps. Potential research targets can be identified by highlighting the genes that have undergone the greatest alteration. **(A)** A volcano plot illustrates the up- and down-regulated gene regulation for the GSE24206 IPF dataset. **(B)** A volcano map illustrates the gene regulation (up and down) in COPD accession GSE76925. **(C)** The volcano graphic, which illustrates gene regulation, uses the context of the GSE18842 lung cancer dataset to display both upregulated and downregulated genes.

The GSE24206 dataset comprised 23 samples that were included in the analysis, and it was discovered that these samples had IPF infection. The gene expression levels of the top 20 genes derived from the chosen samples are presented in Fig 2(A). Furthermore, gene expression characteristics are provided for all samples, including fat tissue and healthy controls. This set includes patient samples that are insulin-resistant and patient samples that show insulin sensitivity. The expression levels of the top 20 genes in the GSE76925 dataset are illustrated in Fig 2(B). Among them 111 samples of COPD and healthy controls, gene expression characterization is also described in detail for 23 samples, including 8 healthy controls. The distinctions between COPD observations and healthy controls clarify the several COPD observation categories, as seen in Fig 2(B). In a similar vein, Fig 2(C) shows the comparison of LC data to healthy controls, highlighting the various categories of LC observations. When comparing LC samples with normal samples for the GSE18842 dataset, up-regulated and down-regulated genes are shown in a volcano plot in Fig 3(A, B, and C), taking into account an adjusted P-value <0.05.

### 3.2 Identification of DEGs and the genetic interaction among IPF, COPD, and Lung cancer

**Fig 4:**
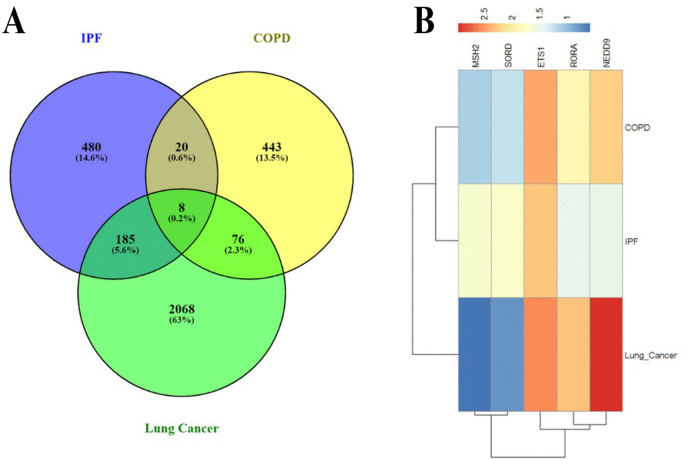
(A) The diagram named venn depicts the overlap of DEGs identified across the three conditions. The GSE24206, GSE76925, and GSE18842 datasets were discovered to share eight DEGs through the identification of shared genes. The possible use of similar genes as biomarkers or targets for treatment is emphasised by this analysis, which is further supported by their regular variation in expression through a variety of lung diseases. **(B)** A heat map visualisation of the genes common among these three conditions. The heatmap was produced by log-fold transforming the top four DEGs, which are frequently common among the datasets for lung cancer, IPF, and COPD.

Using R programming, we analyzed the gene expressions for IPF, which involves 693 genes, lung cancer, which involves 2338 genes, and COPD, which involves 597 genes. 8 common Differentially Expressed Genes (DEGs) were found, as shown in Fig 4(A). Furthermore, a specialized analysis centered on IPF found 11 DEGs that were shared by all the datasets. Using RNA-seq and microarray analysis in R, 2338 genes were identified as being associated with COPD, IPF, and lung cancer, of which 293 were up-regulated and 891 down-regulated. Using the DESeq2 and limma software programs for analysis, a comparable method found significant DEGs for COPD and IPF. The Fig 4(B) is the heat map of the shared genes among these three diseases. By log fold modifying the top 5 consistently shared Differentially Expressed Genes (DEGs) among the lung cancer, IPF, and COPD datasets, a heat map was created. A clearer understanding of the relationships and consequences of the respiratory conditions under research is made possible by the discovery of common and unique gene expressions.

### 3.3 ML-method for gene selections

After identifying the DEGs from the GSE24206, GSE76925, and GSE18842 datasets, we employed the mRMR algorithm to prioritize the most informative genes while minimizing redundancy. mRMR was independently applied to the DEGs of each dataset, and the top 1,500 genes were selected based on relevance and non-redundancy. To further refine this gene subset, we applied SVM-RFE to the mRMR-ranked genes. This two-step selection process yielded the top 1,000 genes from each dataset. These genes, identified through ML-based feature selection, were designated as ML-DEGs for subsequent integrative analysis.

### 3.4 Finding of gene ontology (GO) and pathways, along with the analysis of gene set enrichment

**Fig 5:**
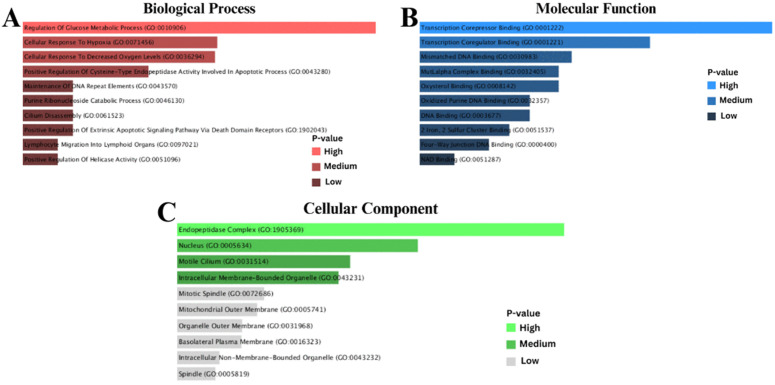
(A) Biological processes: The analysis illustrates that DEGs were categorised into relevant biological processes using Gene Ontology (GO) analysis. The focus of this figure is on the functions that DEGs play in pathways such as immune response, metabolism, and regulation of genes. **(B)** Molecular processes: This analysis utilised Gene Ontology (GO) research and showed that DEGs have a key role in molecular processes such as protein binding, catalytic activity, and signal transduction. **(C)** Cellular component: This figure illustrates that DEGs were categorised by cellular components through Gene Ontology (GO) analysis, which highlighted their distribution in structures such as the plasma membrane, cytoplasm, and nucleus.

**Fig 6:**
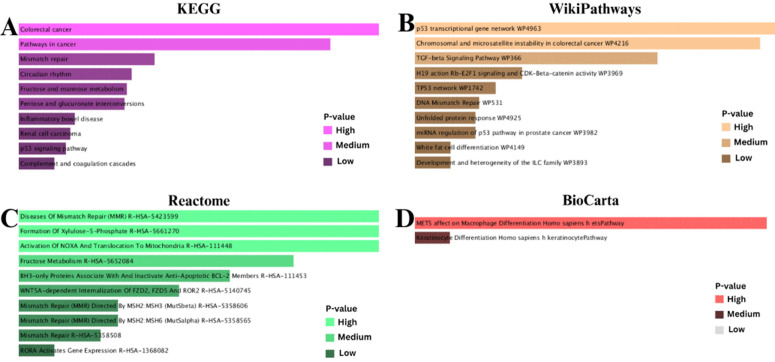
(A) KEGG pathway analysis: Using P-values, the KEGG pathway analysis acknowledged crucial biological pathways that included DEGs, some of which are key signalling pathways and metabolic functions. **(B)** WikiPathways analysis: This is a representation of the WikiPathways analysis that acknowledged the significant pathways associated with the DEGs using biological processes and disease pathways that are significantly represented. **(C)** Reactome pathway analysis: This analysis acknowledges that there are key pathways affected by the DEGs, with a focus on the immune system and signalling functions. **(D)** BioCarta pathway analysis: Using P-values, the pathway analysis acknowledged the increased signalling functions of the DEGs, which provides information on cell functions and disease mechanisms.

The study applied various databases namely WikiPathways, KEGG, BioCarta, and Reactome to detect functionally significant terms and cellular processes. The goal was to find differentially expressed genes (DEGs) that are common among IPF, COPD, and lung cancer. Genes with variable expression showed a substantial correlation with immunity in lung cancer, according to GO and KEGG studies. Gene Ontology analysis gave us a complete picture of all these DEGs’ numerous roles by applying them to BP, CC, and MF. The most impactful 10 BP, cellular components, and molecular activities identified in the GO keywords are listed in Table 2. Then, Enrichr was used to make combined scores that included log z-scores and p-values. This helped researchers figure out exactly how comparable molecular processes work. Because of this method, a lot of research had to be done on the biological parts and functional traits of these DEGs. Since pathway analysis was performed using databases such as KEGG, WikiPathways, Reactome, and BioCarta, it was possible to present a simplified and intricate connection of different diseases and their impact on one another. Analysis using Enrichr and its calculation of P values and z-scores made it possible for a deeper and comprehensive look at GO keywords and pathways, which were essential in understanding intricate biological processes behind different disorders examined. With the usage of DEGs, it was possible to determine and identify essential pathways. These pathways have been presented in Table 3. After a keen examination of well-chosen databases, it was possible to identify IL-17 signaling pathway, Malaria, and Spinal Cord Injury WP2431 as a few of such essential pathways. It was possible to have a clear understanding of Gene Ontology (GO), which was demonstrated in Gene Ontology of Fig 5 (A, B, and C). On the other hand, a clear understanding of a comparison of pathways was achieved using different databases and can be demonstrated in Fig 6 (A, B, C, and D). This provides a clear understanding of how different pathways interlink.

**Table 2:** Moreover, in addition to the list of P-values, an in-depth investigation was conducted in relation to the association between common proteins as well as proteins linked with Gene Ontology terms and Gene Ontology (GO) networks. The investigation carried out in the study also identified enrichment in terms of biological processes, cellular components, and molecular functions that are linked with the genes of importance that were identified in the process. The P-values shown are evidence in statistics that highlight the key roles of key GO keywords and pathways that signify the importance of the (molecular) processes that lie behind them.

Category	GO ID	Term	P-value	Genes
**Biological Process (BP)**	GO:0070098	chemokine-mediated signaling pathway	2.12e-06	CXCL8; CXCL12; CXCL1; CCL18
	GO:1990869	cellular response to chemokine	2.81e-06	CXCL8; CXCL12; CXCL1; CCL18
	GO:0031328	positive regulation of cellular biosynthetic process	1.08e-05	CXCL8; IL1B; TYRP1; HBB; CD36
	GO:0045429	positive regulation of nitric oxide biosynthetic process	1.17e-05	IL1B; HBB; CD36
	GO:1904407	positive regulation of nitric oxide metabolic process	1.30e-05	IL1B; HBB; CD36
	GO:0045428	regulation of nitric oxide biosynthetic process	3.88e-05	IL1B; HBB; CD36
	GO:1904645	response to amyloid-beta	5.18e-05	MMP12; MMP13; CD36
	GO:0070486	leukocyte aggregation	7.35e-05	IL1B; RAC2
	GO:0060353	regulation of cell adhesion molecule production	9.44e-05	CXCL8; IL1B
	GO:0043112	receptor metabolic process	1.19e-04	CXCL8; CD36; NSG1
**Molecular Function (MF)**	GO:0008009	chemokine activity	9.54e-07	CXCL8; CXCL12; CXCL1; CCL18
	GO:0042379	chemokine receptor binding	1.34e-06	CXCL8; CXCL12; CXCL1; CCL18
	GO:0045236	CXCR chemokine receptor binding	2.74e-06	CXCL8; CXCL12; CXCL1
	GO:0005125	cytokine activity	8.91e-06	CXCL8; CXCL12; IL1B; CXCL1; CCL18
	GO:0005509	calcium ion binding	2.46e-04	MMP12; MMP13; DSG1; DSC1; DSC2
	GO:0046872	metal ion binding	1.48e-03	MMP12; MMP13; DSG1; DSC1; DSC2
	GO:0004222	metalloendopeptidase activity	8.07e-03	MMP12; MMP13
	GO:0008517	folic acid transmembrane transporter activity	0.00822	SLC19A2
	GO:0031721	hemoglobin alpha binding	0.00822	HBB
	GO:0086083	cell adhesive protein binding involved in bundle of His cell–Purkinje myocyte communication	0.00822	DSC2
**Cellular Component (CC)**	GO:0001533	cornified envelope	7.24e-07	DSG1; KRT10; DSC1; DSC2
	GO:0030057	desmosome	2.74e-06	DSG1; DSC1; DSC2
	GO:0070820	tertiary granule	1.48e-04	HBB; DSG1; CXCL1; DSC1
	GO:0030666	endocytic vesicle membrane	2.22e-03	TYRP1; RAC2; CD36
	GO:0101002	ficolin-1-rich granule	3.41e-03	HBB; DSG1; DSC1
	GO:1904724	tertiary granule lumen	3.71e-03	HBB; CXCL1
	GO:0101003	ficolin-1-rich granule membrane	4.55e-03	DSG1; DSC1
	GO:0005884	actin filament	6.28e-03	RAC2; COBL
	GO:0099513	polymeric cytoskeletal fiber	8.52e-03	RAC2; COBL; KRT10
	GO:0005587	collagen type IV trimer	9.86e-03	COL4A1

**Table 3:** The researchers then did a thorough analysis to find the significance of the association between the P-values and shared genes in the KEGG, WikiPathways, Reactome, and BioCarta databases. This current study found some significant biological processes that support disease mechanisms by emphasising pathways with high numbers of shared genes. Such pathways may help in the development of the disease itself, where the P-values can serve as significant supportive evidence for these pathways’ roles in the disease’s development. Further study on these pathways can help in finding genetic interactions for the pathogenesis of the disease itself.

Database	Pathway	P-value	Genes
KEGG	Malaria	1.79e-08	CXCL8; IL1B; HBB; HBA2; CD36
	Chemokine signaling pathway	1.48e-05	CXCL8; CXCL12; RAC2; CXCL1; CCL18
	Rheumatoid arthritis	1.62e-05	CXCL8; CXCL12; IL1B; CXCL1
	IL-17 signaling pathway	1.69e-05	CXCL8; MMP13; IL1B; CXCL1
	Viral protein interaction with cytokine and cytokine receptor	2.15e-05	CXCL8; CXCL12; CXCL1; CCL18
	Amoebiasis	2.33e-05	CXCL8; COL4A1; IL1B; CXCL1
	NF-kappa B signaling pathway	2.51e-05	CXCL8; CXCL12; IL1B; CXCL1
	African trypanosomiasis	3.06e-05	IL1B; HBB; HBA2
	Yersinia infection	7.39e-05	CXCL8; IL1B; RAC2; PIP5K1B
	Legionellosis	1.13e-04	CXCL8; IL1B; CXCL1
WikiPathways	Spinal Cord Injury (WP2431)	3.61e-08	MMP12; BTG2; CXCL8; COL4A1; IL1B; CXCL1
	IL-18 signaling pathway (WP4754)	4.87e-06	BTG2; CXCL8; MMP13; IL1B; CD36; CCL18
	SARS-CoV-2 innate immunity evasion (WP5039)	1.74e-04	CXCL8; CXCL12; CXCL1
	COVID-19 adverse outcome pathway (WP4891)	2.74e-04	CXCL8; IL1B
	Allograft Rejection (WP2328)	4.22e-04	CXCL8; CXCL12; IL1B
	LTF danger signal response (WP4478)	4.44e-04	CXCL8; IL1B
	Overview of nanoparticle effects (WP3287)	4.44e-04	CXCL8; COL4A1
	Senescence and Autophagy in Cancer (WP615)	6.84e-04	CXCL8; IL1B; CXCL1
	Cytokines and Inflammatory Response (WP530)	8.37e-04	IL1B; CXCL1
	RAS and bradykinin pathways in COVID-19 (WP4969)	1.04e-03	CYP24A1; IL1B
Reactome	Interleukin-10 Signaling (R-HSA-6783783)	5.54e-05	CXCL8; IL1B; CXCL1
	Chemokine Receptors Bind Chemokines (R-HSA-380108)	1.07e-04	CXCL8; CXCL12; CXCL1
	Interleukin-4 and Interleukin-13 Signaling (R-HSA-6785807)	7.23e-04	CXCL8; IL1B; CD36
	Collagen Degradation (R-HSA-1442490)	1.98e-03	MMP12; MMP13
	Binding and Uptake of Ligands by Scavenger Receptors (R-HSA-2173782)	2.08e-03	COL4A1; CD36
	Assembly of Collagen Fibrils and Other Multimeric Structures (R-HSA-2022090)	3.98e-03	MMP13; COL4A1
	Peptide Ligand-Binding Receptors (R-HSA-375276)	4.07e-03	CXCL8; CXCL12; CXCL1
	Keratinization (R-HSA-6805567)	4.81e-03	KRT19; DSG1; KRT10
	Cytochrome P450 - Arranged by Substrate Type (R-HSA-211897)	5.15e-03	CYP24A1; CYP3A5
	Innate Immune System (R-HSA-168249)	6.31e-03	MMP12; IL1B; RAC2; DSG1; CXCL1; CD36
BioCarta	Hemoglobin’s Chaperone (h_ahspPathway)	2.04e-04	HBB; HBA2
	NF*κ*/B activation by Nontypeable *H. influenzae* (h_nthiPathway)	1.04e-03	CXCL8; IL1B
	TSP-1 Induced Apoptosis in Endothelial Cells (h_tsp1Pathway)	1.15e-02	CD36
	CCR5 Signaling in Macrophages (h_Ccr5Pathway)	1.48e-02	CXCL12
	BTG Family Proteins and Cell Cycle Regulation (h_btg2Pathway)	1.48e-02	BTG2
	G-Protein Signaling Through Tubby Proteins (h_tubbyPathway)	1.64e-02	CXCL12
	CXCR4 Signaling Pathway (h_cxcr4Pathway)	1.80e-02	CXCL12
	Regulators of Bone Mineralization (h_npp1Pathway)	1.80e-02	COL4A1
	Activation of PKC through G-protein Coupled Receptors (h_pkcPathway)	1.80e-02	CXCL12
	Platelet Amyloid Precursor Protein Pathway (h_plateletAppPathway)	2.29e-02	COL4A1

### 3.5 PPIs network for hub gene discovery

**Fig 7:**
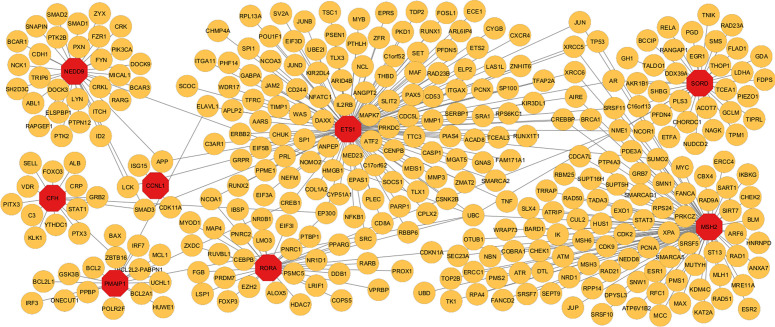
This figure represents PPI network analysis among three diseases, which provides a total of eight genes mutual to all three diseases. All of these were identified as differentially expressed genes. Among these genes are CFH, MSH2, SORD, NEDD9, CCNL1, RORA, ETS1, and PMAIP1. The above genes show prominent interaction as well as shared biological roles, which are also known to play an important role within the pathophysiology of the conditions listed above. This study brings the spotlight on the varied interaction patterns that these genes demonstrate with each other, thus allowing them to play a crucial part in the development of these conditions through these interaction pathways. In this study, understanding these interaction patterns will help scientists gain insights into these cellular events, thus allowing the identification of further targets for research-driven treatment options for these conditions. Using these networks, researchers can thus create better and more functional assessments of shared DEGs, thereby establishing a basis for developing more targeted treatment options.

The PPI network was constructed from the DEGs using NetworkAnalyst and the STRING tool. The Protein-Protein Interaction (PPI) network analysis done using the Cytoscape software in Fig 7 with 95 nodes and 94 edges, revealed the four main hub genes as ETS1, MSH2, RORA, and PMAIP1. The four hub genes play an important role in that they can act as markers and drugs for the diseases IPF and lung cancer. The sub-network was generated to elucidate the relationship between the four hub genes in terms of their interactions and closeness. The entire exercise done in this research by expanding the PPI network correlates with the study intending to discover medicinal drugs for the diseases.

### 3.6 Using topological analysis, the PPI network’s, and identified hub nodes

**Fig 8:**
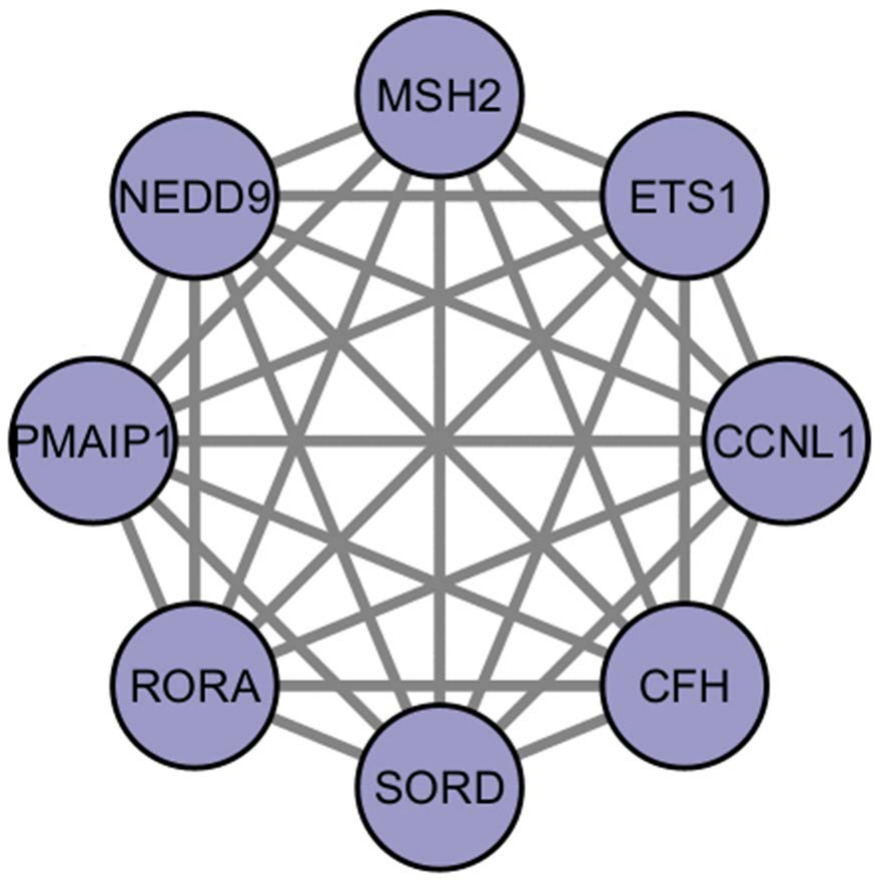
The figure illustrate how hub genes are identified from the common differentially expressed genes across the three given conditions. (ETS1 and MSH2) and (RORA and PMAIP1) are significant nodes. This network highlights the importance these genes have in the whole network. Our significant degree of connection clearly indicates how important their roles are in basic pathways, and that is why they act as hubs. Identifying these genes at once shows their function and how they can be used as variables, as they are important in this network.

In order to investigate the biological significance of network PPI, the identification of hub genes has been performed by means of CytoHubba, a tool integrated into Cytoscape. Among the highly impactful genes, selected for their high degree values, which means broad interaction in the PPI network, such genes as ETS1, MSH2, RORA, and PMAIP1 were found. Specific locations of the PPI network where these hub nodes are located are considered crucial modules. The network of identification of the hub node contains 64 nodes and 63 edges, as represented in Fig 8. Such modules, like a highly connected node, may act as crucial hub genes; hence, therapeutic research and our knowledge about disease pathways might be remarkably improved. In this connection, research on hub genes was underlined as significant for the revealing of sophisticated network structure and for the identification of possible biomarkers for targeted therapeutic approaches. The hub genes have immense value for the application of therapeutics and diagnostics for COPD, IPF, and LC. MSH2 has significance for genomic stability and cancer. RORA has implications for the regulation of fibrosis and immunoregulations. ETS1, NEDD9, and SORD imply the progression of cancer, while SORD has significance for the regulation of metabolism. The parameters that have been used for topological studies identified with the four widely acknowledged hub genes, as depicted above in Table 4, present significant details for the estimation of their functions, indicating that all of them have implications that relate to or affect the network. In the same way, using this technique, we could attempt to establish the significance of the complex cases of diseases by highlighting the importance of the hub genes in the process of discovering the special therapy in which everything revolves.

**Table 4:** Based on topological outcomes, the top four hub genes that are identified as ETS1, MSH2, RORA, and PMAIP1 have been given below. The results indicate that these genes play a significant role in the network and suggest that they have great potential for therapeutic applications. The topological outcomes have helped identify that these genes are significant components of MCODE networks due to their high interaction levels within protein-protein interaction networks, indicating their great potential for therapeutic applications. This data not only reveals the structural significance of these parts but also emphasises the great potential for utilising them as markers and therapeutic targets in the field of health.

*Hub gene*	*Degree*	*Stress*	*Closeness Centrality*	*Betweenness Centrality*
ETS1	133	394372.0	204.16667	72812.13461
MSH2	95	305904.0	176.46667	51293.2645
RORA	34	59786.0	137.65	19531.40852
PMAIP1	33	48032.0	137.5	19569.67527

### 3.7 Validation of the identified hub genes

#### 3.7.1 Diagnostic potential evaluation of the hub genes.

**Fig 9:**
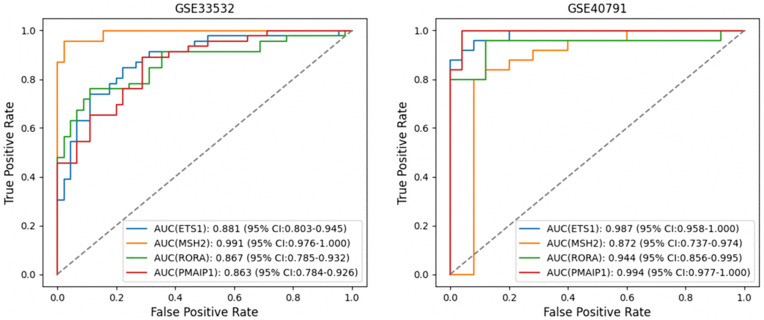
A comprehensive assessment of the diagnostic capabilities of the candidate genes was performed using ROC curve analysis. **(a)** ROC curves depicting the performance of the common hub genes in the GSE33532 dataset; **(b)** ROC curves for the same genes in the GSE40791 dataset. The findings highlight these genes’ strong capability to differentiate tumour samples from normal ones, reinforcing their potential as reliable diagnostic biomarkers.

The performance of the hub genes was tested for diagnosis using receiver operating characteristic (ROC) curve analysis on the two separate validation datasets, GSE33532 and GSE40791. The hub genes had a high discriminative power for diagnosis in GSE33532, as the AUC for all genes was above 0.95. The highest discriminative power was found for the MSH2 gene (AUC = 0.991), followed by ETS1 (AUC = 0.881), RORA (AUC = 0.867), and PMAIP1 (AUC = 0.863) for distinguishing lung cancer tissues from normal tissues. Similar observations were made for GSE40791, where the gene PMAIP1 had the highest discriminative power (AUC = 0.994), followed by ETS1 (AUC = 0.987), RORA (AUC = 0.944), and MSH2 (AUC = 0.872) for distinguishing lung cancers from normal tissues. The high AUC values for the hub genes in the two datasets indicate the applicability of the hub genes as a discriminative marker for the diagnosis of lung cancers. The corresponding curves are shown in Fig 9.

#### 3.7.2 Survival analysis.

**Fig 10:**
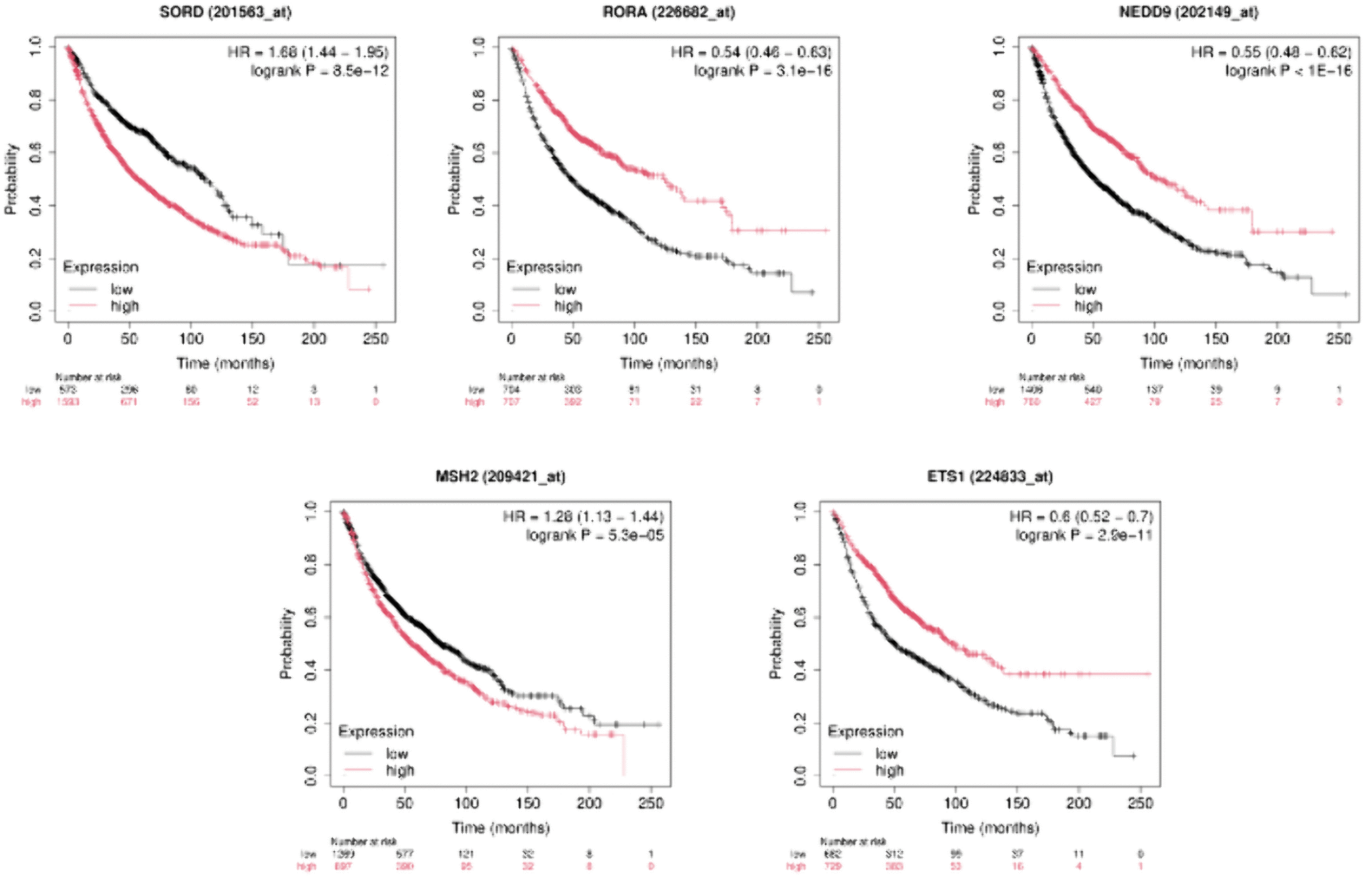
Survival analysis of key genes, including ETS1, MSH2, RORA, and PMAIP1, has been generated by the product limit (PL) estimator with the study of common DEGs in IPF, COPD, and LC. The red colour denotes normal expression, blue underexpression, and green overexpression with these graphs. People with modified gene expression have a lower rate of survival compared to those with normal gene expression, as demonstrated vividly by the survival variances between the two groups. Concerning these results, ETS1, MSH2, RORA, and PMAIP1 might be significant biomarkers to evaluate patient outcomes in disease diagnosis. These particular genes ought to be the focus of further research to figure out the roles they play in diseases and to create targeted treatments, considering the reported survival imbalances indicate that their function is essential.

In the above research, the authors employed the survival technique in their attempt to understand and extract essential gene expressions linked with Chronic Obstructive Pulmonary Disease (COPD), Lung Cancer, and idiopathic pulmonary fibrosis (IPF). Survival values were calculated in the different groups, whereby the differences in modified and normal gene expression were determined using the Cox PH model and Product Limit (PL) estimator. In determining the genes that are most adversely affected, we apply the Cox PH regression model in either univariate or multivariate testing at a p-value of < 0.05. The Fig 10 shows the survival rates for the important genes ETS1, MSH2, RORA, and PMAIP1 in the PL estimator. The survival rates for the individuals having the transformed gene expression are lower than those of individuals having normal expression. This can be identified by the graphs. The visualization of the significance levels of gene expression on survival, as illustrated in the graphs, shows normal expression as the red line, underexpression as the blue line, and overexpression as the green line. After performing survival analysis, we got altered expression of the hub genes that can help in personalized treatment planning and potential risk stratification. The results of survival analysis also suggest the value of monitoring disease progression, aiding in early diagnosis, and guiding treatment response. The understanding obtained by the current research will prove beneficial when organizing further research and the development of specific therapies for lung cancer, chronic obstructive pulmonary disease (COPD), and idiopathic pulmonary fibrosis (IPF).

### 3.8 Tf-gene Interactions

**Fig 11:**
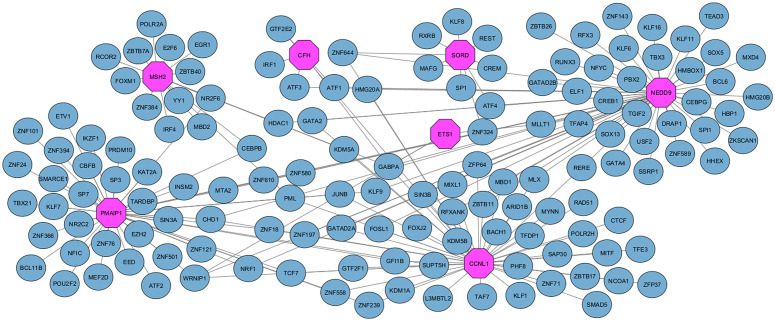
This figure illustrates tf-gene interaction, which is based on common DEGs; nodes that are pink in colour denote common DEGs, whereas nodes that are light green represent tf-genes. The aforementioned depiction additionally shows significant regulatory pathways related to disease generation, but it also shows the regulatory connections between transcription factors, as well as the particular genes that are addressed. By discovering these correlations, the network reveals mechanisms involved in transcriptional regulation and therapeutic strategies. By assigning each node a different colour for analysis, it becomes possible to distinguish which DEGs are relevant and which TFs are involved in them; this process reveals the correlation between these factors, as well as disease mechanisms.

In order to construct the TF-gene interactions, NetworkAnalyst has been utilized. Common Differentially Expressed Genes (DEGs) such as CFH, ETS1, CCNL1, NEDD9, MSH2, RORA, PMAIP1, and SORD have been identified as TF-genes. The Fig 11 depicts the connections between commonly employed DEGs and TF controllers. The total number of 95 nodes and 94 edges contributes to the network.

### 3.9 TF-miRNA co-regulatory network

**Fig 12:**
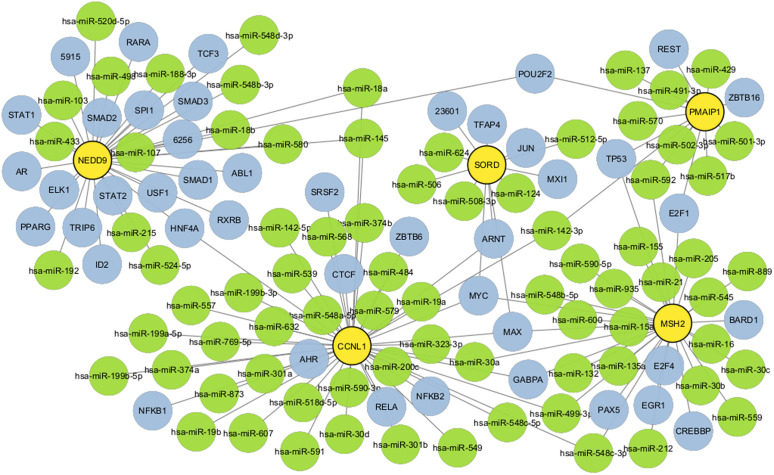
This figure shows the TF-miRNA regulation network, which has been coloured to identify the responsibilities of the nodes: the yellow nodes represent miRNAs, the red nodes denote common differentially expressed genes (DEGs), and the remaining nodes correspond to the other DEGs. The dynamic regulatory networks composed of transcriptional factors (TFs), microRNAs (miRNAs), and DEGs are depicted by colours in this diagram. By demonstrating each of these relationships in turn, the network narrows its focus to significant miRNAs, which may serve as powerful regulators, controlling the gene expression of various DEGs concurrently. Understanding such regulatory interactions is very essential for identifying drug targets and developing means of modifying gene activity in disease contexts. The network sets the ground to uncover miRNAs that execute an extensive range of regulatory activities that may be a useful tool in searching for specific therapies directed toward the regeneration of appropriate gene expression profiles in disease.

The TF-miRNA coregulatory network, which was developed utilizing NetworkAnalyst, provides an in-depth knowledge of the regulatory relationships within transcription factors (TFs) and microRNAs (miRNAs) through the use of common differentially expressed genes (DEGs). The structure of this network, which consists of 101 nodes and 131 edges, demonstrates the ability for transcription factors (TFs) and miRNAs to jointly affect the expression of DEGs, consequently potentially regulating gene activity and modifying disease pathways. In this network, 39 miRNAs and 53 TF-genes interact by way of common DEGs, revealing a complex regulatory layer in which both TFs and miRNAs combine to regulate gene expression profiles. The key regulatory interactions that are evidenced by the structure and interaction in the above network, as shown in Fig 12, may help in the treatment of the condition by concentrating on these interactions of TF and miRNAs.

### 3.10 Drug compound identification

The drug molecules have been extracted from the database named DSigDB using the freely accessible tool. In accordance with the p-value and adjusted p-value, subsequent treatments were expected. The table that follows includes common DEGs that can be used as therapeutic targets for idiopathic pulmonary fibrosis (IPF), chronic obstructive pulmonary disease (COPD), and lung cancer. In this analysis drug-gene relation is primarily based on the adjusted p-values and combined scores. Then the reported roles are considered in modulating and fibrosis, as well as safety profiles for potential repurposing. The identified drug components show a strong link with the hub genes that have documented mechanisms, such as anti-inflammatory and anti-fibrosis, relevant to LC, COPD, and LC. This selection also gives a practical translational way where existing agents could be repositioned for lung disease treatment. Table 5 shows the largest number of possible medicinal compounds determined by the most widely used DEGs.

**Table 5:** By analysing the shared DEGs, molecules related to IPF, COPD, and lung cancer have been detected based on drug suggestions. By this method, new pharmacologic agents have been recognised for targeting shared DEGs as potential treatments. These new pharmacologic agents have provided promising results in curing different types of lung diseases. The verification stage and drug development process will be explored in identifying molecules based on the new pharmacologic agents. By identifying common biological features, this approach promises to develop more efficient, multi-conditional, wide-spectrum therapies.

*Name of drugs*	*P-value*	*Adjusted P-value*	*Genes*
astemizole MCF7 UP	0.0000345	0.009510477	NEDD9; PMAIP1; CCNL1
calmidazolium MCF7 UP	0.0000401	0.009510477	NEDD9; PMAIP1; CCNL1
ivermectin MCF7 UP	0.0000926	0.009510477	NEDD9; PMAIP1
DICHLOROMETHANE CTD 00006313	0.0001631	0.009510477	NEDD9; PMAIP1
ETHYLBENZENE CTD 00000178	0.0001699	0.009510477	NEDD9; PMAIP1
O-XYLENE CTD 00001228	0.0001698	0.009510476	PMAIP1
digoxigenin PC3 UP	0.0001700	0.009510476	NEDD9; PMAIP1; CCNL1
ethaverine HL60 DOWN	0.0001982	0.009510476	NEDD9; PMAIP1
N-Acetyl-L-cysteine CTD 00005305	0.0002043	0.009510476	NEDD9; PMAIP1; ETS1
8-azaguanine PC3 UP	0.0002200	0.009510476	NEDD9; PMAIP1; CCNL1

## 4 Discussion

This research combines transcriptomics information with machine learning approaches guided by systems biology to decode shared molecular signatures in Idiopathic Pulmonary Fibrosis, Chronic Obstructive Pulmonary Disease, and Lung Cancer, which are highly connected and represent a significant proportion of the global morbidity and mortality cases. The convergent findings for four crucial hub genes, ETS1, MSH2, RORA, and PMAIP1, give insights into possible mechanistic correlations between chronic inflammation, remodelling, and carcinogenesis within the lung microenvironment [62,63].

Eight common DEGs co-exist in the GSE24206 (IPF), GSE18842 (LC), and GSE76925 (COPD) microarray datasets, suggesting that general mechanisms of chronic inflammatory signalling and immune abnormalities contribute to the pathogenesis of all three diseases. In addition, enrichment analysis of DEGs focused on chemokine-mediated signalling transduction, IL-17, NF, C-C chemokine receptor interactions, Cytokine-Cytokine receptor interaction, etc., of great pathogenesis significance in inflammatory cascade events underlying epitheliogenesis, carcinogenesis, and fibrosis. All of the identified core genes contribute in their own way toward maintaining genomic stability. By mRMR-SVM RFE analysis, this study overcame the limitations imposed by gene redundancies in gene expression profiling analysis. The study combined topological properties of the PPI network with co-regulating properties of regulatory elements in the TF-miRNA regulatory network, identifying network-regulated events ETS1-miR200-RORA regulatory axis and MSH2-AR-PMAIP1 regulatory axis relevant in epitheliogenesis and apoptosis events, respectively. This novel combination analysis approach overcomes the limitations associated with individual single-omics research methods in the biological sciences.

The high accuracy (AUC > 0.85) of the independent datasets emphasises the value of these genes as universal biomarkers for various diseases. The common nature of their deregulation points towards the possibility of using liquid biopsy panels in identifying high-risk patients with COPD/IPF for their possible neoplastic change. Moreover, enrichment analysis using drug-gene association led to the identification of several candidates like astemizole, ivermectin, and N-acetyl-L-cysteine, which were previously shown to possess anti-inflammatory and antioxidant effects and could instead be targeted for the modulation of pathways involving ETS1 and PMAIP1.

Previous studies have shown transcriptomic similarity between IPF and LC and between COPD and LC. Our results confirm these and expand upon them in that we used a cross-disease machine learning approach in which convergent expressions are quantified in terms of survival prediction. The finding of DNA repair/apoptosis genes as core network hubs is consistent with a “fibrosis-to-cancer axis” because of the known concept that cellular stress leads both to fibrosis and cancer development. While the utilisation of public microarray data improves generalisation, the study is compromised in that it is comprised of highly variable population samples with small numbers of IPF patients and is not verified at the protein or single-cell levels. This study primarily aimed to identify transcriptomic alterations shared across IPF, COPD, and lung cancer, rather than mutation-driven oncogenic subtypes. Due to the lack of uniform somatic mutation and stage-level clinical metadata in public datasets, survival analysis was performed using expression-based stratification, which partially reflects disease aggressiveness and patient prognosis despite the absence of formal staging information. The absence of uniform COPD severity grading (e.g., GOLD stage or FEV1) across public datasets limited stratified severity analysis; however, the identified transcriptomic signatures consistently reflect disease-associated molecular alterations.

Finally, this is an integrated study that highlights the strong linkage among IPF, COPD, and LC from the molecular perspective as conditions with shared inflammation networks as well as genomic instability networks. Finally, the hub genes proposed in this study may play the roles of diagnostic warning indicators as well as therapeutic targets for multi-target drug discovery approaches.

## 5 Conclusion

Through applying a transcriptome analysis, this research offers information on currently unexplored common molecular biomarkers and pathways among LC, COPD, and IPF. Through significant bioinformatics tools, this paper proved some critical Differentially Expressed Genes (DEGs) in these mentioned respiratory diseases by using three microarray datasets across many studies (GSE24206, GSE18842, GSE76925). Through the construction of Protein-Protein Interaction (PPI) networks and determination with shared DEGs, hub genes ETS1, MSH2, RORA, and PMAIP1 were identified, which are important for various diseases. The study serves as groundwork to identify potential therapeutic targets and biomarkers for diagnosis. This study identifies the shared genes among IPF, COPD, and LC from a different perspective, allowing us to gain new insights into these three diseases and to locate potential therapeutic targets and biomarkers for disease management. This study also begins to explain why people with diseases such as IPF and COPD are at increased risk of developing lung cancer by showing that common responses to infection may also be key determinants of whether or not a person is likely to develop this disease. It shows the importance of more thorough research into lung cancer, especially its subtypes because of their heterogeneous aetiology, and opens an opportunity for transcriptome analysis in seeking common pathways that might be valuable therapeutic targets. The detection of four hub genes which are associated with the progression of disease and their correlation with functional mutations, as well as regulatory elements such as TFs or miRNAs, helped us to open a new perspective for developing specific drugs, even a lung cancer vaccine. In this study, we discuss the importance of understanding multiple interactions among IPF and COPD with lung cancer for implications on future drugs to develop novel therapeutic strategies or prophylaxis. In total, this thorough exploration forms a basis for future studies to identify common molecular pathways in different respiratory conditions and will aid us in discovering new treatments. In summary, we believe that our comprehensive analysis is a solid resource for further in-depth exploration of common stress response pathways in respiratory conditions and a basis for discovering new multi-target therapy.

## References

[1] Fathinavid A, Mousavian Z, Najafi A, Nematzadeh S, Salimi M, Masoudi-Nejad A. Identifying common signatures and potential therapeutic biomarkers in COPD and lung cancer using miRNA-mRNA co-expression networks. Inform Med Unlocked. 2022;34:101115. doi: 10.1016/j.imu.2022.101115

[2] Dai Z-T, Wang J, Zhao K, Xiang Y, Li JP, Zhang H-M, et al. Integrated TCGA and GEO analysis showed that SMAD7 is an independent prognostic factor for lung adenocarcinoma. Medicine (Baltimore). 2020;99(44):e22861. doi: 10.1097/MD.0000000000022861 33126329 PMC7598801

[3] LoPiccolo J, Gusev A, Christiani DC, Jänne PA. Lung cancer in patients who have never smoked - an emerging disease. Nat Rev Clin Oncol. 2024;21(2):121–46. doi: 10.1038/s41571-023-00844-0 38195910 PMC11014425

[4] Chiavarini M, Rosignoli P, Sorbara B, Giacchetta I, Fabiani R. Benzene Exposure and Lung Cancer Risk: A Systematic Review and Meta-Analysis of Human Studies. Int J Environ Res Public Health. 2024;21(2):205. doi: 10.3390/ijerph21020205 38397694 PMC10887806

[5] Jemal A, Bray F, Center MM, Ferlay J, Ward E, Forman D. Global cancer statistics. CA Cancer J Clin. 2011;61(2):69–90. doi: 10.3322/caac.20107 21296855

[6] Yang IA, Relan V, Wright CM, Davidson MR, Sriram KB, Savarimuthu Francis SM, et al. Common pathogenic mechanisms and pathways in the development of COPD and lung cancer. Expert Opin Ther Targets. 2011;15(4):439–56. doi: 10.1517/14728222.2011.555400 21284573

[7] Hosen MdF, Basar MdA, Yasmin MstF, Hasan MdR, Uddin MS. Identify the potential pathways and candidate biomarkers of stroke associated with bipolar disorder: Bioinformatics and system biology approach. J Proteins Proteom. 2025;16(2):141–59. doi: 10.1007/s42485-025-00181-z

[8] Kotlyarov S. The Role of Smoking in the Mechanisms of Development of Chronic Obstructive Pulmonary Disease and Atherosclerosis. Int J Mol Sci. 2023;24(10):8725. doi: 10.3390/ijms24108725 37240069 PMC10217854

[9] Safiri S, et al. Burden of chronic obstructive pulmonary disease and its attributable risk factors in 204 countries and territories, 1990-2019: results from the global burden of disease study 2019. BMJ. 2022;378. 10.1136/bmj-2021-069679 PMC9326843 35896191

[10] Park SC, Kim DW, Park EC, Shin CS, Rhee CK, Kang YA, et al. Mortality of patients with chronic obstructive pulmonary disease: a nationwide populationbased cohort study. Korean J Intern Med. 2019;34(6):1272–8. doi: 10.3904/kjim.2017.428 31610634 PMC6823577

[11] Song Q, Chen P, Liu X-M. The role of cigarette smoke-induced pulmonary vascular endothelial cell apoptosis in COPD. Respir Res. 2021;22(1):39. doi: 10.1186/s12931-021-01630-1 33546691 PMC7866753

[12] Giezeman M, Sundh J, Athlin Å, Lisspers K, Ställberg B, Janson C, et al. Comorbid Heart Disease in Patients with COPD is Associated with Increased Hospitalization and Mortality - A 15-Year Follow-Up. Int J Chron Obstruct Pulmon Dis. 2023;18:11–21. doi: 10.2147/COPD.S378979 36644219 PMC9838124

[13] Finkelstein J, Cha E, Scharf SM. Chronic obstructive pulmonary disease as an independent risk factor for cardiovascular morbidity. Int J Chron Obstruct Pulmon Dis. 2009;4:337–49. doi: 10.2147/copd.s6400 19802349 PMC2754086

[14] Sidney S, Sorel M, Quesenberry CP Jr, DeLuise C, Lanes S, Eisner MD. COPD and incident cardiovascular disease hospitalizations and mortality: Kaiser Permanente Medical Care Program. Chest. 2005;128(4):2068–75. doi: 10.1378/chest.128.4.2068 16236856

[15] Cavaillès A, Brinchault-Rabin G, Dixmier A, Goupil F, Gut-Gobert C, Marchand-Adam S, et al. Comorbidities of COPD. Eur Respir Rev. 2013;22(130):454–75. doi: 10.1183/09059180.00008612 24293462 PMC9639181

[16] Divo M, Cote C, de Torres JP, Casanova C, Marin JM, Pinto-Plata V, et al. Comorbidities and risk of mortality in patients with chronic obstructive pulmonary disease. Am J Respir Crit Care Med. 2012;186(2):155–61. doi: 10.1164/rccm.201201-0034OC 22561964

[17] Gagnat AA, Gjerdevik M, Lie SA, Gulsvik A, Bakke P, Nielsen R. Acute exacerbations of COPD and risk of lung cancer in COPD patients with and without a history of asthma. Eur Clin Respir J. 2020;7(1):1799540. doi: 10.1080/20018525.2020.1799540 32944202 PMC7480432

[18] Hosen MdF, Basar MdA, Paul BK, Hasan MdR, Uddin MS. A bioinformatics approach to identify candidate biomarkers and common pathways between bipolar disorder and stroke. In: 2022 12th International Conference on Electrical and Computer Engineering (ICECE). 2022. p. 429–32.

[19] Young RP, Hopkins R, Eaton TE. Forced expiratory volume in one second: not just a lung function test but a marker of premature death from all causes. Eur Respir J. 2007;30(4):616–22. doi: 10.1183/09031936.00021707 17906084

[20] Maher TM, Bendstrup E, Dron L, Langley J, Smith G, Khalid JM, et al. Global incidence and prevalence of idiopathic pulmonary fibrosis. Respir Res. 2021;22(1):197. doi: 10.1186/s12931-021-01791-z 34233665 PMC8261998

[21] Herazo-Maya JD, Kaminski N. Personalized medicine: applying “omics” to lung fibrosis. Biomark Med. 2012;6(4):529–40. doi: 10.2217/bmm.12.38 22917154 PMC3517740

[22] Tzouvelekis A, Herazo-Maya J, Sakamoto K, Bouros D. Biomarkers in the Evaluation and Management of Idiopathic Pulmonary Fibrosis. Curr Top Med Chem. 2016;16(14):1587–98. doi: 10.2174/1568026616666150930120959 26420365

[23] Spagnolo P, Sverzellati N, Rossi G, Cavazza A, Tzouvelekis A, Crestani B, et al. Idiopathic pulmonary fibrosis: an update. Ann Med. 2015;47(1):15–27. doi: 10.3109/07853890.2014.982165 25613170

[24] Rosas IO, Kaminski N. Update in diffuse parenchymal lung disease, 2013. Am J Respir Crit Care Med. 2015;191(3):270–4. doi: 10.1164/rccm.201405-0856UP 25635490 PMC4351573

[25] Allen RJ, Stockwell A, Oldham JM, Guillen-Guio B, Schwartz DA, Maher TM, et al. Genome-wide association study across five cohorts identifies five novel loci associated with idiopathic pulmonary fibrosis. Thorax. 2022;77(8):829–33. doi: 10.1136/thoraxjnl-2021-218577 35688625 PMC9329250

[26] Partanen JJ, Häppölä P, Zhou W, Lehisto AA, Ainola M, Sutinen E, et al. Leveraging global multi-ancestry meta-analysis in the study of idiopathic pulmonary fibrosis genetics. Cell Genom. 2022;2(10):100181. doi: 10.1016/j.xgen.2022.100181 36777997 PMC9903787

[27] Ghosh AJ, Hobbs BD, Yun JH, Saferali A, Moll M, Xu Z, et al. Lung tissue shows divergent gene expression between chronic obstructive pulmonary disease and idiopathic pulmonary fibrosis. Respir Res. 2022;23(1):97. doi: 10.1186/s12931-022-02013-w 35449067 PMC9026726

[28] Basar MA, et al. Identification of drug and protein-protein interaction network among stress and depression: A bioinformatics approach. Inform Med Unlocked. 2023;37:101174.

[29] Leng D, Yi J, Xiang M, Zhao H, Zhang Y. Identification of common signatures in idiopathic pulmonary fibrosis and lung cancer using gene expression modeling. BMC Cancer. 2020;20(1):986. doi: 10.1186/s12885-020-07494-w 33046043 PMC7552373

[30] Vella D, Marini S, Vitali F, Di Silvestre D, Mauri G, Bellazzi R. MTGO: PPI Network Analysis Via Topological and Functional Module Identification. Sci Rep. 2018;8(1):5499. doi: 10.1038/s41598-018-23672-0 29615773 PMC5882952

[31] Szklarczyk D, Morris JH, Cook H, Kuhn M, Wyder S, Simonovic M, et al. The STRING database in 2017: quality-controlled protein-protein association networks, made broadly accessible. Nucleic Acids Res. 2017;45(D1):D362–8. doi: 10.1093/nar/gkw937 27924014 PMC5210637

[32] Li N, Qiu L, Zeng C, Fang Z, Chen S, Song X, et al. Bioinformatic analysis of differentially expressed genes and pathways in idiopathic pulmonary fibrosis. Ann Transl Med. 2021;9(18):1459. doi: 10.21037/atm-21-4224 34734011 PMC8506768

[33] Dasgupta S. Identification and molecular modelling of potential drugs targeting the genes involved in the progression of lung cancer in patients with idiopathic pulmonary fibrosis. Gene Rep. 2024;33:102067. doi: 10.1016/j.genrep.2024.102067

[34] Basar MA, Hasan MR, Paul BK, Shadhin KA, Mollah MS. A system biology and bioinformatics approach to determine the molecular signature, core ontologies, functional pathways, drug compounds in between stress and type 2 diabetes. In: International Work-Conference on Bioinformatics and Biomedical Engineering, 320–331 (Springer, 2023).

[35] Barrett T, Suzek TO, Troup DB, Wilhite SE, Ngau W-C, Ledoux P, et al. NCBI GEO: mining millions of expression profiles--database and tools. Nucleic Acids Res. 2005;33(Database issue):D562–6. doi: 10.1093/nar/gki022 15608262 PMC539976

[36] Sarker S, Hosen MdF, Bashar MA, Ahammed E. Integrated Bioinformatics and Machine Learning Analysis Reveals Shared Key Candidate Biomarkers and Therapeutic Targets in Ulcerative Colitis and Colorectal Cancer. In: 2024 2nd International Conference on Information and Communication Technology (ICICT). 2024. p. 105–9.

[37] Emid E, et al. Gene symbol gene title logfc p value (< 0.01). J Name.

[38] Singh RK, Sivabalakrishnan M. Feature Selection of Gene Expression Data for Cancer Classification: A Review. Procedia Computer Science. 2015;50:52–7. doi: 10.1016/j.procs.2015.04.060

[39] Lazar C, Taminau J, Meganck S, Steenhoff D, Coletta A, Molter C, et al. A survey on filter techniques for feature selection in gene expression microarray analysis. IEEE/ACM Trans Comput Biol Bioinform. 2012;9(4):1106–19. doi: 10.1109/TCBB.2012.33 22350210

[40] Chen JW, Dhahbi J. Lung adenocarcinoma and lung squamous cell carcinoma cancer classification, biomarker identification, and gene expression analysis using overlapping feature selection methods. Sci Rep. 2021;11(1):13323. doi: 10.1038/s41598-021-92725-8 34172784 PMC8233431

[41] Li W, Liu J, Zhu W, Jin X, Yang Z, Gao W, et al. Identification of biomarkers for hepatocellular carcinoma based on single cell sequencing and machine learning algorithms. Front Genet. 2022;13:873218. doi: 10.3389/fgene.2022.873218 36353113 PMC9638064

[42] Sanz H, Valim C, Vegas E, Oller JM, Reverter F. SVM-RFE: selection and visualization of the most relevant features through non-linear kernels. BMC Bioinform. 2018;19(1):432. doi: 10.1186/s12859-018-2451-4 30453885 PMC6245920

[43] Rakotomamonjy A. Variable selection using svm-based criteria. J Mach Learn Res. 2003;3:1357–70.

[44] Consortium GO. Expansion of the gene ontology knowledgebase and resources. Nucleic Acids Res. 2017;45:D331–8. 10.1093/nar/gkw1108 PMC5210579 27899567

[45] Wittig U, De Beuckelaer A. Analysis and comparison of metabolic pathway databases. Brief Bioinform. 2001;2(2):126–42. doi: 10.1093/bib/2.2.126 11465731

[46] Doms A, Schroeder M. GoPubMed: exploring PubMed with the Gene Ontology. Nucleic Acids Res. 2005;33(Web Server issue):W783–6. doi: 10.1093/nar/gki470 15980585 PMC1160231

[47] Slenter DN, Kutmon M, Hanspers K, Riutta A, Windsor J, Nunes N, et al. WikiPathways: a multifaceted pathway database bridging metabolomics to other omics research. Nucleic Acids Res. 2018;46(D1):D661–7. doi: 10.1093/nar/gkx1064 29136241 PMC5753270

[48] Fabregat A, Jupe S, Matthews L, Sidiropoulos K, Gillespie M, Garapati P, et al. The Reactome Pathway Knowledgebase. Nucleic Acids Res. 2018;46(D1):D649–55. doi: 10.1093/nar/gkx1132 29145629 PMC5753187

[49] Nishimura D. Biocarta. Biotech Softw Internet Report. 2001;2:117–20.

[50] Kanehisa M, Sato Y, Kawashima M, Furumichi M, Tanabe M. KEGG as a reference resource for gene and protein annotation. Nucleic Acids Res. 2016;44(D1):D457–62. doi: 10.1093/nar/gkv1070 26476454 PMC4702792

[51] Kanehisa M, Goto S. KEGG: kyoto encyclopedia of genes and genomes. Nucleic Acids Res. 2000;28(1):27–30. doi: 10.1093/nar/28.1.27 10592173 PMC102409

[52] Kuleshov MV, Jones MR, Rouillard AD, Fernandez NF, Duan Q, Wang Z, et al. Enrichr: a comprehensive gene set enrichment analysis web server 2016 update. Nucleic Acids Res. 2016;44(W1):W90–7. doi: 10.1093/nar/gkw377 27141961 PMC4987924

[53] Hosen MdF, Basar MdA, Yasmin MstF, Morshed M, Uddin MS. Identification of Key Signaling Pathways and Novel Computational Drug Target for Depression and Coronary Artery Disease. In: 2024 IEEE International Conference on Computing, Applications and Systems (COMPAS). 2024. p. 1–4.

[54] Xia J, Gill EE, Hancock REW. NetworkAnalyst for statistical, visual and network-based meta-analysis of gene expression data. Nat Protoc. 2015;10(6):823–44. doi: 10.1038/nprot.2015.052 25950236

[55] Shannon P, Markiel A, Ozier O, Baliga NS, Wang JT, Ramage D, et al. Cytoscape: a software environment for integrated models of biomolecular interaction networks. Genome Res. 2003;13(11):2498–504. doi: 10.1101/gr.1239303 14597658 PMC403769

[56] Chin C-H, Chen S-H, Wu H-H, Ho C-W, Ko M-T, Lin C-Y. cytoHubba: identifying hub objects and sub-networks from complex interactome. BMC Syst Biol. 2014;8(Suppl 4):S11. doi: 10.1186/1752-0509-8-S4-S11 25521941 PMC4290687

[57] Berezka KM, Kovalchuk OYa, Banakh SV, Zlyvko SV, Hrechaniuk R. A Binary Logistic Regression Model for Support Decision Making in Criminal Justice. Folia Oeconomica Stetinensia. 2022;22(1):1–17. doi: 10.2478/foli-2022-0001

[58] Khan A, et al. Jaspar 2018: update of the open-access database of transcription factor binding profiles and its web framework. Nucleic Acids Research. 2018;46:D260–6. 10.1093/nar/gkx1126 PMC5753243 29140473

[59] Liu Z-P, Wu C, Miao H, Wu H. RegNetwork: an integrated database of transcriptional and post-transcriptional regulatory networks in human and mouse. Database. 2015;2015:bav095. doi: 10.1093/database/bav095 PMC4589691 26424082

[60] Hsu S-D, Lin F-M, Wu W-Y, Liang C, Huang W-C, Chan W-L, et al. miRTarBase: a database curates experimentally validated microRNA-target interactions. Nucleic Acids Res. 2011;39(Database issue):D163–9. doi: 10.1093/nar/gkq1107 21071411 PMC3013699

[61] Yoo M, Shin J, Kim J, Ryall KA, Lee K, Lee S, et al. DSigDB: drug signatures database for gene set analysis. Bioinformatics. 2015;31(18):3069–71. doi: 10.1093/bioinformatics/btv313 25990557 PMC4668778

[62] Wen S, Peng W, Chen Y, Du X, Xia J, Shen B, et al. Four differentially expressed genes can predict prognosis and microenvironment immune infiltration in lung cancer: a study based on data from the GEO. BMC Cancer. 2022;22(1):193. doi: 10.1186/s12885-022-09296-8 35184748 PMC8859904

[63] Wu Z, Chen H, Ke S, Mo L, Qiu M, Zhu G, et al. Identifying potential biomarkers of idiopathic pulmonary fibrosis through machine learning analysis. Sci Rep. 2023;13(1):16559. doi: 10.1038/s41598-023-43834-z 37783761 PMC10545744

